# Taking a closer look: Can an app improve diagnostic accuracy in urgent care? Cluster-randomized interventional trial DASI

**DOI:** 10.1371/journal.pdig.0001252

**Published:** 2026-02-24

**Authors:** Eva Maria Noack, Kai Antweiler, Tim Friede, Frank Müller, Tobias Schmidt, Eva Hummers, Lea Roddewig, Dominik Schröder

**Affiliations:** 1 Department of General Practice, University Medical Center Göttingen, Göttingen, Lower Saxony, Germany; 2 Department of Medical Statistics, University Medical Center Göttingen, Göttingen, Lower Saxony, Germany; 3 Department of Family Medicine, Michigan State University, Grand Rapids, Michigan, United State of America; 4 Medical School Hamburg, University of Applied Sciences and Medical University, Hamburg, Hamburg, Germany; Yonsei University College of Medicine, KOREA, REPUBLIC OF

## Abstract

In urgent care settings, efficient medical history-taking is paramount for making timely and accurate treatment decisions. Medical history-taking apps have emerged as a means to streamline this process but their effectiveness in enhancing diagnostic accuracy remains unclear. We aimed to investigate whether using a medical history-taking app before consultation improves diagnostic accuracy. In two German out-of-hours practices (OOHP), patients were recruited over a 12-months period. Within each practice, weeks were randomized to either an intervention or control group, resulting in a cluster-randomized trial (CRT) with clustering in weeks within the same practice. Patients in the intervention group used an app to report their complaints before their consultation, enabling physicians to review their medical history details beforehand. In contrast, patients in the control group used the app after their consultation, and no summary of their medical history was available to the physician. Diagnostic accuracy was defined as the agreement between the OOHP physician’s diagnoses and those determined by an expert committee (EC) after reviewing patient files. As a secondary outcome, we compared OOHP and EC physicians’ treatment recommendations against patients’ self-reported actual treatment (e.g., specialist care, hospital admissions) from a follow-up survey. We analyzed data from 986 patients and found no significant intervention effect on diagnostic accuracy (Odds Ratio 0.94 (95%CI 0.73 – 1.21), 57.6% in intervention vs 59.1% in control group). Additionally, the app had no significant effect on the prediction of further treatment. The only significant factors affecting these outcomes were the number of diagnoses (positively associated with diagnostic accuracy) and a self-reported severe condition (associated with higher likelihood of requiring further treatment). Individual differences between physicians were more pronounced than those between the intervention and control group for the secondary outcome. The study’s findings suggest that this medical history-taking app does not enhance diagnostic accuracy in urgent care settings.

## Author summary

Medical history-taking apps allow patients to report their symptoms and related information before seeing a doctor, which can help doctors make more informed decisions. However, it remains unclear whether these apps enhance the accuracy of diagnoses. To investigate this, we compared the accuracy of diagnoses made with and without the use of a medical history-taking app in a setting where timely and accurate diagnoses are particularly crucial. The study took place in two out-of-hour practices, characterized by limited time and diagnostic capacities, and with changing staff with different medical specialties. In most encounters, physicians and patients met for the first time, making it a challenging environment for diagnosis. Our assumption was that the app could be particularly valuable in this setting, as it could provide physicians with essential information before the consultation. However, our findings suggest that the app did not improve the accuracy of diagnoses. This study contributes to our understanding of the potential benefits and limitations of medical history-taking apps. Although our app did not enhance diagnostic accuracy, it may still be useful in other ways, such as helping patients prepare for their appointments, providing doctors with additional information to consider or reducing th documentation burden. Further research could explore these potential benefits of medical history apps to improve patient care in urgent care settings.

## Background

In 2022, a total of 17.6 million out-of-hour urgent care visits were counted across Germany, with 10.3 million treated in the emergency departments and 7.3 million in walk-in out-of-hour practices (OOHP) [1,2]. Unlike in general practice, where patients are established with a practice or a physician and receive continuous care, in urgent care situations physicians frequently treat unfamiliar patients. OOHPs are staffed by rotating physicians from practices within the district, representing various specialties (e.g.,., general practice, internal medicine, dermatology, gynecology and obstetrics, otolaryngology, and neurology) and experience levels. Given OOHPs’ limited diagnostic facilities, accurate diagnosis and management rely heavily on patient interviews and physical examinations. In internal medicine and general practice clinics, correct diagnosis can mostly be made based on a thorough medical history [3,4]. However, OOHP physicians often face considerable workload, resulting in time constraints for medical history-taking which would be crucial for accurate diagnosis and appropriate treatment [5].

Several medical history-taking apps have been designed to support physicians in gathering information on current health problems [6–11], but only a few are commercially available. These apps offer various features, such as customizable questionnaires, decision support algorithms, multilingual support, or integration with electronic medical records (EMR). Medical history-taking apps have potential in streamlining workflows, aiming to optimize the collection of patient data, to improve documentation accuracy, and to enhance patient engagement [12–15]. Despite medical history-taking apps’ proposed benefits, research on their impact is limited: while usability studies are common, evaluations of their effect on patient care and health outcomes remain sparse [16].

This paper reports on a study evaluating the impact of a newly developed urgent care medical history-taking app on diagnostic accuracy. The used app, referred to as “DASI-app”, was developed as a tool for structured symptom-oriented medical history-taking. The app’s usability and the quality of the collected information were addressed in earlier studies [17–19]. In this study, we are testing the following hypotheses:

1) The use of the DASI-app prior to consultation in OOHP improves diagnostic accuracy.
2) The use of the DASI-app impacts the physician’s recommendation for further treatment after an OOHP consultation.

## Materials and methods

### Study design

This study was carried out in two centers. In each center, one-week periods were randomized to intervention or control before data collection started. Therefore, all patients recruited in a practice within the same week belong to the same intervention group. Cluster randomization in one-week periods was chosen to minimize the risk of contamination. In each week, one practice was in the intervention group, another in the control group. As randomization was carried out in two-week blocks, the practices were in the same group for a maximum of two consecutive weeks. The random allocation sequence was generated by a member of the study team not involved in the data collection. Recruitment continued for 12 consecutive months (03/01/2022 – 02/28/2023). The primary outcome was the diagnostic accuracy. The study was open, i.e., patients and treating physicians were not blinded to the assigned intervention. Detailed information about the methodology can be found in the study protocol [20]. The study was conducted according to the guidelines of the Declaration of Helsinki and approved by the Institutional Review Board of the University Medical Center Göttingen (approval no. 26/3/21). The study is registered in the German register for clinical trials (DRKS00026659). Study methods and results are reported following the ‘Consolidated Standards of Reporting Trials (CONSORT) 2010 statement: extension to cluster randomized trials’ [21].

### Intervention: The medical history-taking app ‘DASI’

**Fig 1:**
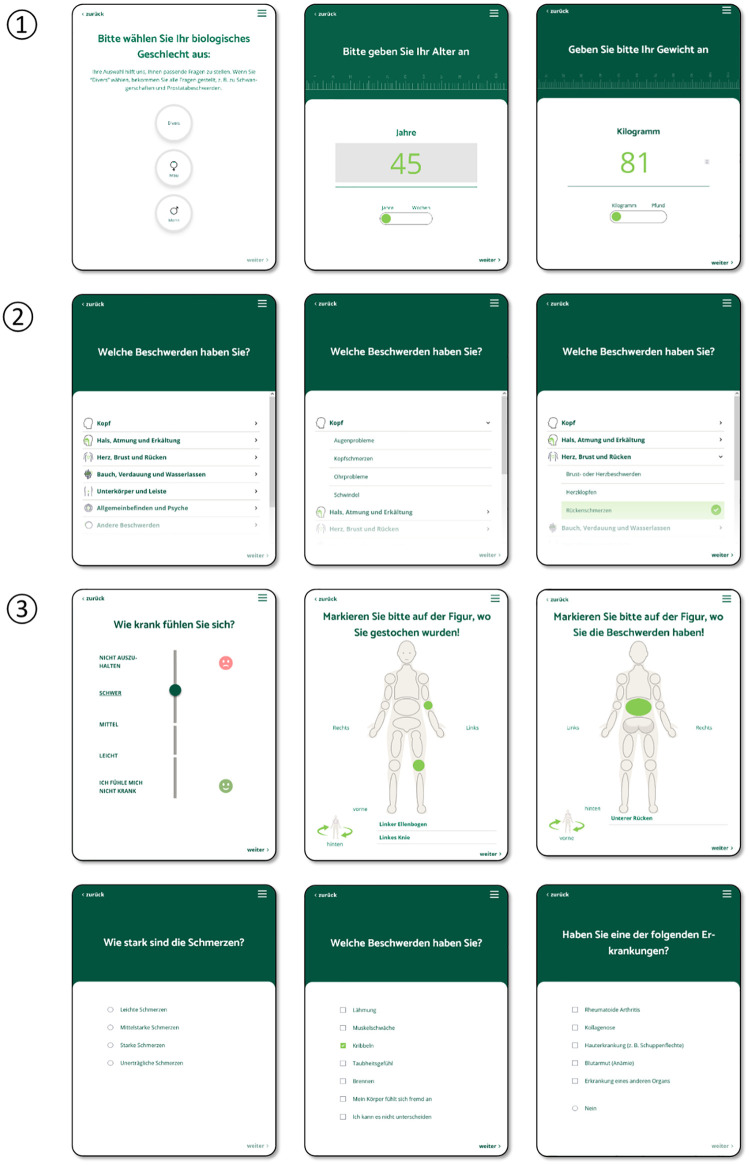
Screenshots of the app for medical history-taking in general practice. ① demographic information with data entry field for age and weight; ② Choice of complaints; ③ Types of response options: slider for questions including a ranking between items, body figure indicator, single choice question, multiple choice question, and hybrid question, i.e., one can either select several options or negate all of them.

**Fig 2:**
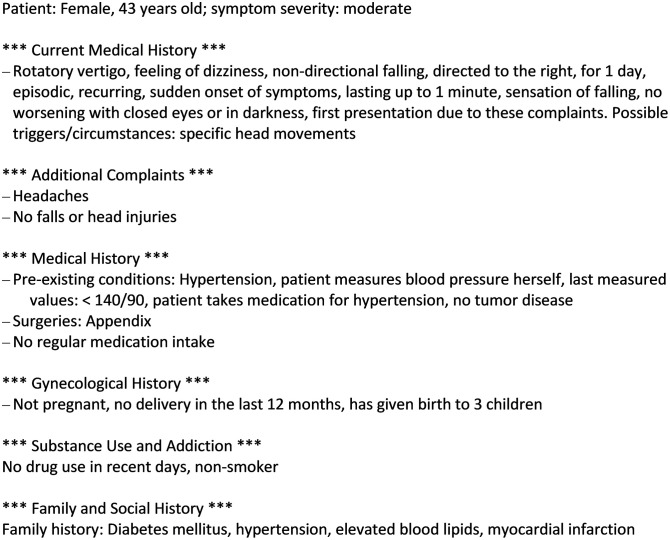
Exemplary report of a short medical history taken by the app (translated into English by the authors).

The DASI-app is intended to be used by patients in the waiting room prior to the encounter with the physician and is designed for patients with acute but not life-threatening conditions that are typical for primary care encounters. After providing information on gender, age, height, and weight, patients are asked to select one or more complaints from a variety of the most prevalent acute health concerns in urgent care. Based on the selected complaint(s), they receive follow-up questions as part of a dynamic questionnaire, i.e., their responses may trigger further specific questions related to the chosen complaint(s). Patients may also be asked about pre-existing conditions, previous treatments and medical procedures, current drug prescriptions, lifestyle habits, and family history of chronic conditions. The app uses various response types: single and multiple-choice selections, data entry fields, sliders, and body figure indicators (Fig 1). The app uses plain language, making it accessible to people with low health literacy. An earlier study confirmed that the app is intuitive to use without prior instruction [18]. After completing the query, the app generates a structured medical history report, which can be transferred to the patient’s EMR for physician review prior to the encounter. The app does not synthesize/interpret data into recommendations or suggestions for diagnoses. Instead, this report displays the patient’s self-reported information in an aggregated and structured manner using concise and clear medical language (e.g., (e.g., “hypertension” instead of “high blood pressure”, “fluid leakage from the ear” instead of “fluid comes out of the ear” (Fig 2). For this purpose, all response options were tagged with keywords that define their information content. A simple example: ‘right side’ or ‘leg’ can describe a ‘localization’ (e.g., of an injury) but ‘leg’ can also be used to describe the place to where a pain radiates. ‘When coughing’ can be used to describe a (pain) trigger or a factor that aggravates a pain.

For this study, the DASI-app generated a data matrix QR code containing the patient’s medical history report. Medical assistants at the reception desk scanned these QR codes to transfer the information into the EMR. The transferred information appeared as structured text in the free-text medical history field of the patient’s electronic record. The app supports the medical history-taking in several languages; the medical history report can be generated in German or English. For this study, only the German-language interface was used.

### Setting and OOHP selection

The study was carried out in two OOHP in the German Federal State of Lower Saxony that provide urgent care when general practices are closed, e.g., in the evening and night times, on weekends, and public holidays. Services are covered by mandatory health insurance with no co-payments required. OOHP are operated by the Associations of Statutory Health Insurance Physicians (‘Kassenärztliche Vereinigungen’) in Germany but physicians are not employed at the OOHP. Physicians with registered with health insurance funds are required to take on shifts, rotating from practices within the district. It is possible to switch or take over shifts from other physicians. Physicians are from various specialties (e.g., general practice, internal medicine, dermatology, gynecology and obstetrics, otolaryngology, and neurology) and experience levels. Consequently, in most cases, physician and patient are unknown to each other, and as patient files are not shared between practices, physicians must conduct an initial assessment akin to a ‘new patient visit’. OOHPs focus on acute conditions that require prompt attention but are not life-threatening, bridging the gap between general practices and emergency hospital services. Typical conditions treated in OOHP are gastroenteritis, flu-like infections, urinary tract infection, rashes, cervical spine disorders, hypertensive crisis, insect bites and stings, conjunctivitis, lumbago, pneumonia, falls, and tonsillitis [5]. Cases not treated in OOHPs and instead referred to hospital emergency departments include severe injuries (e.g., fractures) and medical emergencies (e.g., acute coronary syndromes, strokes). The specialties participating in the OOPH service include general practitioners (GPs). In Germany, GPs complete a five-year residency program that includes internal medicine (mostly a minimum of one year), general practice settings (with a minimum of two years), and various elective specialties (such as pediatrics, gynecology, surgery etc.). Furthermore, they can achieve further special competencies (“Zusatzbezeichnung”) similar to training fellowships in the U.S. Their training is designed to produce well-rounded primary care physicians not only capable of serving as patients’ first point of contact within the healthcare system and but providing comprehensive patient care across all age groups, managing preventive medicine, urgent care, and chronic conditions.

The OOHPs were purposively selected to represent different healthcare environments while addressing logistical constraints during the COVID-19 pandemic. Since the Kassenärztliche Vereinigung Niedersachsen (KVN, Association of Statutory Health Insurance Physicians of Lower Saxony) was project partner, only practices in Lower Saxony were considered. The first OOHP was located in Göttingen, a university city of 119,000 inhabitants [22] with a large university medical center and a young demographic (21.3% of residents are 18–30 years, [23]), thus serving a rather young urban population. The second OOHP was situated in Northeim, a small town approximately 20 km north of Göttingen with 29,440 inhabitants [22], located within the Lower Saxony hills region. This OOHP has a rural catchment area of small villages with various demographic characteristics. In Göttingen, the OOHP is located adjacent to the university hospital, in Northeim to a small district hospital. This selection allowed for comparison between urban and rural healthcare settings. Due to travel restrictions implemented during the COVID-19 pandemic, we specifically chose OOHPs in proximity to the conducting department. This pragmatic approach to site selection balanced the need for diverse practice settings with the practical constraints imposed by pandemic-related restrictions.

The study OOHPs were equipped with basic diagnostic equipment such as stethoscopes, otoscopes, blood pressure monitors, ultrasound, electrocardiogram, electronic thermometers, and urine dipstick tests. Blood tests could only be performed in Göttingen. The OOHP were always staffed by one attending physician and either one medical assistant (in Northeim) or one to two medical assistants (in Göttingen), depending on the day of the week and expected patient flow.

Patients are generally treated in the order of their arrival, following a first-come, first-served approach. Only in exceptional circumstances might a patient receive priority treatment, such as when they are in severe distress or patients with suspected infectious pathogens (such as suspected norovirus infection). Otherwise, all patients are registered and treated in the sequence they enter the practice. If it becomes apparent that a case is an emergency and/or requires hospital admission, the patient is promptly referred to the emergency department located in the same building.

### Inclusion and exclusion criteria

Participation in the study was voluntary, with inclusion/exclusion criteria applied based on age, medical condition, and consent. Patients could withdraw from participation without giving a reason at any time. Inclusion criteria included: (a) Patients seeking care in one of the participating OOHPs due to acute medical symptom, (b) aged 18 years or older, (c) able to declare written informed consent to participate in study. Exclusion criteria included (a) Patient’s age < 18 year, (b) patients in an apparent medical emergency, (c) patients who required immediate medical treatment, and (d) patients who were unable to consent, including limited German language proficiency. Potential participants were informed by trained study nurses and needed to declare written consent before study participation.

### Data collection

#### Patient recruitment in the out-of-hour practices.

Patients were recruited for an entire year (03/01/2022 – 02/28/2023). A study nurse approached patients in the waiting room of the two practices. The study nurses received training according to the International Conference on Harmonisation - Good Clinical Practice (ICH GCP). Furthermore, all study nurses received study-specific training on the study procedures, including consent documentation and providing information to potential participants. This training emphasized the importance of ensuring participant comprehension, voluntariness, and the right to withdraw at any time without consequences. Regular supervision, weekly team meetings and quality checks of the consent process were implemented to maintain consistency and adherence to ethical standards throughout the recruitment period.

**Fig 3:**
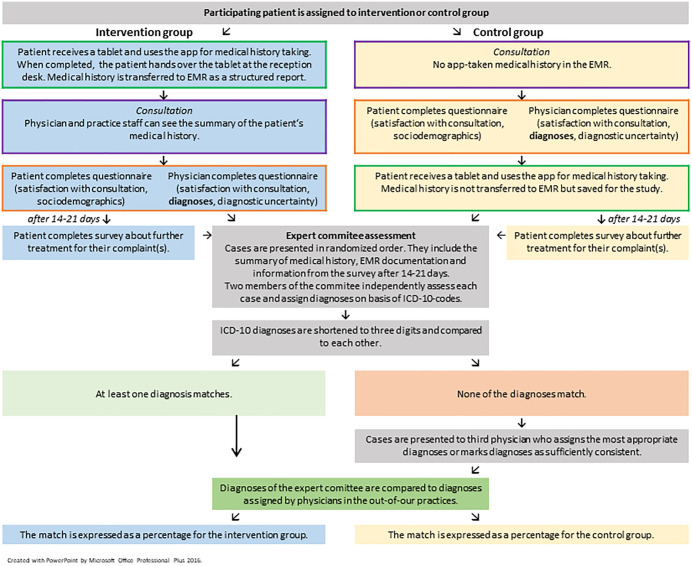
Process of data collection and analysis.

Patients who decided to take part in the study, signed the written informed consent and the privacy policy statement, and provided contact information (email or phone number depending which method of contact they preferred) for a follow-up survey 14–21 days later. Fig 3 visualizes the process of data collection and analysis.

Patients received an iPad mini 5 (Apple Inc., Cuppertino CA) with the DASI-app from study personnel. The intervention group completed the app-based query before the encounter, with information transferred to the EMR for physician use, while the control group used the app post-consultation, providing no pre-visit information to physicians. In both groups, participants were asked about their satisfaction with the consultation and to provide sociodemographic data (current employment status, professional degree, native language). Physicians provided ICD-10-GM (10^th^ revision of the International Statistical Classification of Diseases and Related Health Problems, German Modification) coded diagnoses for each participant, along with recommended next steps (e.g., diagnostic procedures, specialist referrals, hospitalization). They also rated perceived diagnostic uncertainty and consultation satisfaction via a paper-pencil survey. Furthermore, we collected data on physicians’ specializations and work experience, along with their patient documentation for each participant. All data was linked using assigned study identification numbers (IDs) for each patient.

Participants were contacted 14–21 days later via email, text, or phone for a brief follow-up survey about any further treatment (e.g., in practices, clinics, or emergency rooms) for the same complaints. Hospitalized participants were asked for their hospital diagnosis, typically available in their discharge letter. In German primary care settings, patients rarely know their coded diagnosis unless they receive a sick note. Non-responsive patients were contacted up to three times over a 21-day period, after which all contact information were deleted to eliminate links between study IDs and personal data.

#### Expert committee’s assessment to measure diagnostic accuracy.

Our study focused on diagnostic accuracy, defined as the agreement between practicing physicians’ diagnoses and those of a blinded expert committee (EC) serving as the reference standard [24]. Diagnostic accuracy was assessed by comparing OOHP physicians’ diagnoses with those of the EC, composed of three GPs, each with at least 10 years of experience in both general practice and shifts in OOHPs. The members of the EC were selected sequentially based on these criteria and under the condition, that they had not been involved in any previous steps of the DASI-project (e.g., by treating patients at the OOHP in Göttingen or Northeim).

After the full year of data collection in the OOHP was completed, the EC reviewed all cases in a randomized order, blinded to their allocation to intervention or control groups. Each case presented to the EC encompassed patient documentation, including examination findings and the summary of medical history generated by the app yet omitting diagnoses and proposed treatments (e.g., prescribed medication). Furthermore, responses of the participant follow-up survey, collected 14–21 after the encounter, were provided when available. Initially, two EC members (EC1 and EC2) reviewed independently the provided information to formulate ICD-10-GM diagnoses codes. EC members were free to choose the grade of detail of the ICD code with a minimum of a three-digit code. Using a Microsoft Access database (Microsoft Corp., Redmond WA, USA), EC members could browse and search for ICD codes and select up to 10 diagnoses for any respective case. Beyond diagnoses, EC members rated their case assessment confidence and, mirroring OOHP physicians, indicated their diagnostic uncertainty and recommended next steps (e.g., further examinations, specialist referrals, hospitalization) with rationales. The procedure and the functionality of the database were tested in a “validation loop” using 20 cases to make sure the ICD-10 codes were easy to find and to estimate the time required. This task was carried out by an experienced GP who had not been involved in the project before, was thus blinded and not familiar with the database before.

For statistical analysis, the ICD-10-codes were shortened to a three-digit code (e.g., J64.1 to J64). Cases with discrepant diagnoses between EC1 and EC2 were presented to the third expert committee member (EC3) to achieve consensus. This third expert was tasked with selecting the most accurate or probable diagnoses among discordant cases. If applicable, EC3 could mark the discrepant diagnoses by the previous two physicians as sufficiently congruent in a free text field, thereby revealing potential duplications in ICD-10 coding (e.g., R51: ‘headache’ and G44: ‘Other headache syndromes’). In the following analysis, these duplications were then considered as concurrent. Diagnoses identified as sufficiently congruent by the third committee member were also used in the calculation of concordance between EC1 and EC2, as well as in the calculation of diagnostic accuracy between EC and OOHP. Upon identification of overlapping diagnoses between the first two committee members, these were cross-referenced with diagnoses from the OOHP. Subsequently, the diagnoses of EC3 were compared with those from the OOHP.

### Measures

#### Diagnostic accuracy.

Diagnostic accuracy was defined as the match of at least one diagnosis between OOHP physician’s diagnoses and the consensus diagnoses reached by the EC, which served as the reference standard.

#### Accuracy of the recommendation for following treatment.

For the accuracy of physicians’ treatment recommendations (e.g., referrals for specialist care, hospital admissions) we compared OOHP and EC physicians’ recommendations to patients’ self-reported follow-up care, calculating agreement rates in percent.

### Sample size calculation

For the primary outcome, we assumed an increase in diagnostic accuracy of 10 percentage points, based on a current diagnostic accuracy of 70–80% [25,26]. To determine the required sample sizes, a design with randomization of individual patients was considered, which yielded a power of 90% at a two-sided significance level of 5% with 670 patients (335 per group), assuming diagnostic accuracy of 75% and 85%. To further account for cluster randomization, the sample size was multiplied by the design effect, which is determined by cluster size and intraclass correlation (ICC). The calculation assumed a cluster size of 36 patients per 2-week cluster across both practices and an ICC of 0.01. The difference between the two practices was adjusted in the analysis model using a fixed-effect, resulting in a design effect of 1.35. The sample size was further adjusted for an expected dropout rate of 5%. Taking these factors into account, a total sample size of 1,000 patients was determined. Before the beginning of the study, the cluster length was reduced to one week while the number of clusters was doubled. This modification slightly reduced the number of cases needed (originally 952) to 825. Both numbers were rounded to 1,000 patients, thus retaining the original sample size.

### Statistical analysis

Patient characteristics are presented as count and proportion for categorical variables, and median and IQR for continuous variables. The primary outcome diagnostic accuracy was adjusted with a logistic mixed model for study center as fixed effect and the time cluster as random effect. For explorative analyses the patient’s age, number of diagnoses given by the OOHP, number and severity (categorical) of complaints, an indicator variable for “OOHP was a GP”, were added as fixed effect covariates to a second model. Agreement of the OOHP physician’s recommendation of further consultation with the patient actually seeking further consultation was analyzed with a logistic mixed model for study center as fixed effect and the time cluster as random effect. For explorative analyses the patient’s age, number of diagnoses given by the OOHP, number and severity (categorical) of complaints, an indicator variable for “OOHP was a GP”, were again added as fixed effect covariates to a second model. We run subgroup analyses for German as a native language (yes/no), year of state examination (before 1990/1990–2000/after 2000), patient age (<60 years/ ≥ 60 years), number of complaints (1/ ≥  2), severity of complaints, specialty of treating OOHP physician (GP/not GP), and center (Göttingen/Northeim) to explore whether a certain subgroup of patients or physicians would benefit from pre-consultation history-taking. Agreement of the OOHP physician’s recommendation of further consultation with the patient actually seeking further consultation was analyzed with the approach. Agreement rates of recommendations for further treatment of each physician (OOHP and EC) were reported as percentages together with 95%-confidence intervals (according to the Clopper-Pearson method) separately for intervention and control group as well as for the overall study population. Differences between cases that were assessed by the EC and those that were not were tested with Mann-Whitney *U*-Tests for continuous and χ²-Tests for categorical variables. No adjustment for multiple testing was carried out. Descriptive p-values (two-sided) are reported and referred to as statistically significant if they were <0.05. R (version 4.4.1) was used for all computations.

## Results

### Sample characteristics

**Fig 4:**
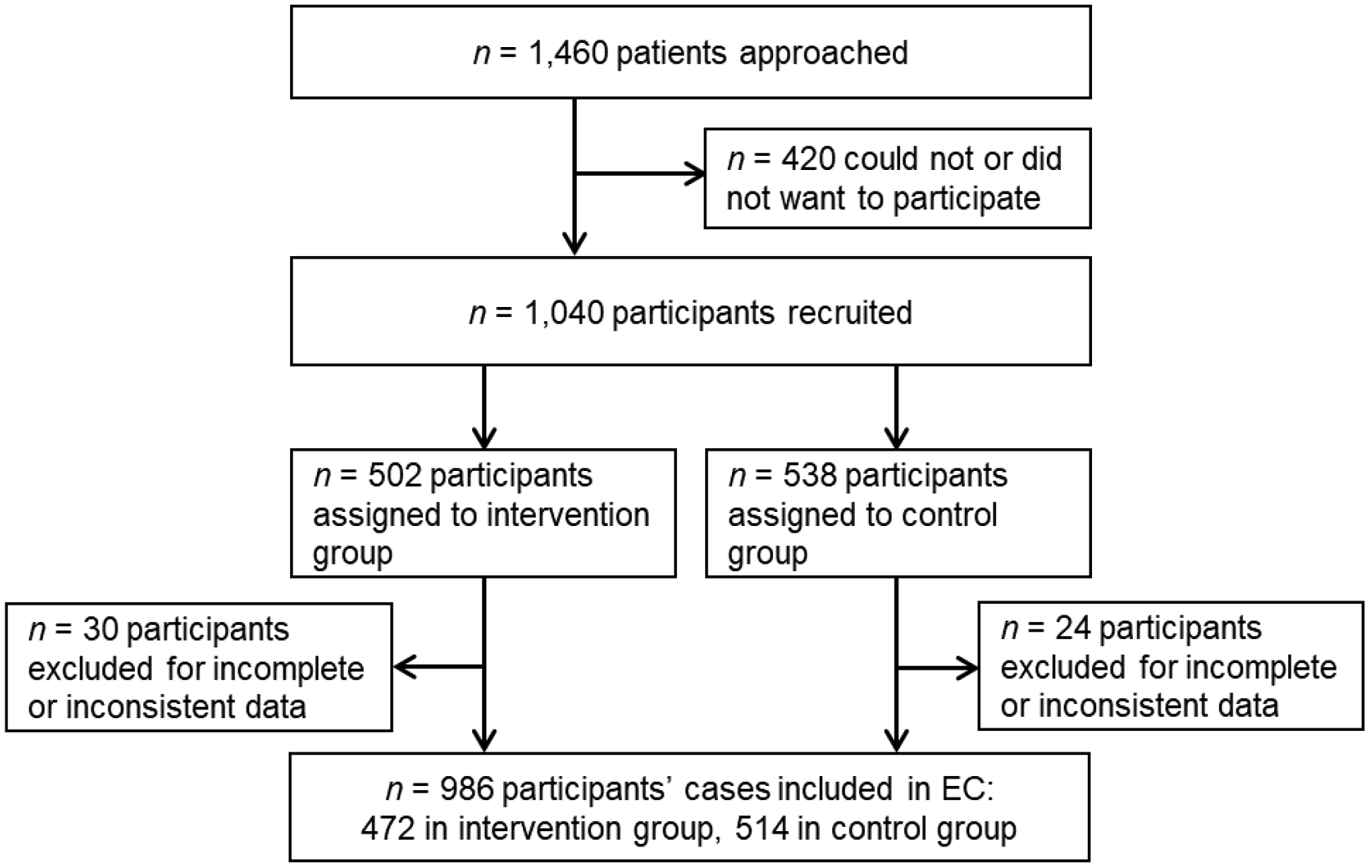
Flowchart. EC: expert committee.

Fig 4 shows the flowchart of included patients. From 1,460 approached patients 1,040 patients were recruited for the study. Of these 1,040 study participants, 54 were excluded from the analyses in this paper (S1 Table), among others because the diagnosis provided by physician was not readable or did not match patient documentation. Reasons for exclusion can be found in S2 Table.

Characteristics of participants are shown in Tables 1 and 2. Participants in the intervention group were older than in the control group (median 32.0 years vs. 30.0 years). Participants recruited in Göttingen were younger than in Northeim (median 29.0 years vs. 34.0 years) and had a lower body weight (74 kg vs. 80 kg). In Göttingen, participants had higher professional qualifications and there were more university students (26.8% vs. 2.2%).

**Table 1:** Characteristics of participants - overall and by intervention and control groups.

		Overall n = 986 (100%)	Intervention group n = 472 (47.9%)	Control group n = 514 (52.1%)
Sex, n (%)	male	384 (38.9)	182 (38.6)	202 (39.3)
	female	602 (61.1)	290 (61.4)	312 (60.7)
Age, median (IQR)	years	31.0 (24.0, 44.0)	32.0 (25.0, 45.0)	30.0 (23.0, 42.8)
Number of diagnoses, median (IQR)	n	1.00 (1.00, 2.00)	1.00 (1.00, 2.00)	1.00 (1.00, 1.00)
Number of complaints, median (IQR)	n	1.0 (1.0, 2.0)	1.0 (1.0, 2.0)	1.00 (1.0, 2.0)
Severity of complaints, n (%)	I don’t feel sick	105 (10.7)	48 (10.2)	57 (11.1)
	Mild	183 (18.6)	81 (17.2)	102 (19.9)
	Medium	488 (49.6)	228 (48.4)	260 (50.8)
	Severe	187 (19.0)	103 (21.9)	84 (16.4)
	Unbearable	20 (2.0)	11 (2.3)	9 (1.8)
Height, median (IQR)	cm	171.0 (165.0, 180.0)	171.0 (165.0, 180.0)	171.0 (165.0, 180.0)
Weight, median (IQR)	kg	77. 0 (65.0, 90.0)	78.0 (65.0, 90.0)	76.0 (65.0, 90.0)
Highest professional qualification, n (%)	Master’s degree/ Diploma/ State examination/ PhD	128 (13.1)	64 (13.8)	64 (12.5)
	Bachelor‘s degree	78 (8.0)	33 (7.1)	45 (8.8)
	Master craftsman/ technician or equivalent	39 (4.0)	20 (4.3)	19 (3.7)
	Completed vocational training	240 (24.6)	113 (24.4)	127 (24.8)
	High school diploma/ Advanced technical college certificate	214 (21.9)	99 (21.4)	115 (22.5)
	Secondary school certificate	168 (17.2)	70 (15.1)	98 (19.1)
	Elementary/lower secondary school certificate	84 (8.6)	52 (11.2)	32 (6.2)
	Other qualifications	11 (1.1)	4 (0.9)	7 (1.4)
	No degree	13 (1.3)	8 (1.7)	5 (1.0)
Current employment status, n (%)	Employed/working	531 (55.5)	251 (55.5)	280 (55.4)
	In vocational training	84 (8.8)	41 (9.1)	43 (8.5)
	University student	154 (16.1)	65 (14.4)	89 (17.6)
	Voluntary/civilian/military service	6 (0.6)	1 (0.2)	5 (1.0)
	Student at school	22 (2.3)	10 (2.2)	12 (2.4)
	Retired	59 (6.2)	31 (6.9)	28 (5.5)
	Unemployed	18 (1.9)	8 (1.8)	10 (2.0)
	other	83 (8.7)	45 (10.0)	38 (7.5)
German as native language, n (%)	Yes	856 (87.9)	398 (86.1)	458 (89.5)
Study center, n (%)	Northeim	445 (45.1)	226 (47.9)	219 (42.6)

**Table 2:** Characteristics of participants - by centers.

		Göttingen n = 541	Northeim n = 445
Sex, n (%)	male	214 (39.6)	170 (38.2)
	female	327 (60.4)	275 (61.8)
Age, median (IQR)	years	29.0 (23.0, 39.0)	34.0 (26.0, 50.0)
Number of diagnoses, median (IQR)	n	1.0 (1.0, 1.0)	1.0 (1.0, 2.0)
Number of complaints, median (IQR)	n	1.0 (1.0, 2.0)	1.00 (1.0, 2.0)
Severity of complaints, n (%)	I don’t feel sick	58 (10.8)	47 (10.6)
	Mild	99 (18.4)	84 (18.9)
	Medium	264 (49.1)	224 (50.3)
	Severe	106 (19.7)	81 (18.2)
	Unbearable	11 (2.0)	9 (2.0)
Height, median (IQR)	cm	172.0 (165.0, 180.0)	170.0 (165.0, 179.5)
Weight, median (IQR)	kg	74.0 (63.0, 88.0)	80.0 (69.0, 95.0)
Highest professional qualification, n (%)	Master’s degree/ Diploma/ State examination/ PhD	91 (16.9)	37 (8.4)
	Bachelor‘s degree	59 (11.0)	19 (4.3)
	Master craftsman/ technician or equivalent	18 (3.4)	21 (4.8)
	Completed vocational training	109 (20.3)	131 (29.9)
	High school diploma/ Advanced technical college certificate	147 (27.4)	67 (15.3)
	Secondary school certificate	71 (13.2)	97 (22.1)
	Elementary/lower secondary school certificate	27 (5.0)	57 (13.0)
	Other qualifications	8 (1.5)	3 (0.7)
	No degree	7 (1.3)	6 (1.4)
Current employment status, n (%)	Employed/working	263 (49.8)	268 (62.5)
	In vocational training	42 (8.0)	42 (9.8)
	University student	144 (27.3)	10 (2.3)
	Voluntary/civilian/military service	5 (0.9)	1 (0.2)
	Student at school	10 (1.9)	12 (2.8)
	Retired	17 (3.2)	42 (9.8)
	Unemployed	8 (1.5)	10 (2.3)
	other	39 (7.4)	44 (10.3)
German as native language, n (%)	Yes	462 (86.0)	394 (90.2)
Group, n (%)	Intervention group	246 (45.5)	226 (50.8)

Most complaints indicated through the DASI-app were sore throat (n = 147), abdominal discomfort (n = 105), cough (n = 105), headache (n = 100), difficulty swallowing (n = 99), back pain (n = 85), urinary discomfort (n = 84). Distribution of complaints is presented in S3 Table. Patients were treated mainly by board certified general practitioners (65%), internal medicine specialists (20%), specialists in psychosomatic medicine and psychotherapy (4.3%), gynecologists (3.9%), surgeons (3.8%), otorhinolaryngologists (1.6%), and neurologists (1.3%) (multiple selection possible). On median, they graduated from medical school in 1996 (IQR: 1992, 2001) and received their specialist certification in 2004 (IQR: 2001, 2008).

After 14–21 days, 694 of 986 successfully contacted participants answered the follow-up questionnaire (response rate: 70.4%); 22 could not be reached due to incorrect contact details. Among respondents, 246 (35.4%) received further treatment for their initial complaints, while 448 (64.6%) did not.

#### Expert committee’s assessment.

In 670 (68.0%) of the 986 cases rated by EC1 and EC2, at least one of the diagnoses matched. The 316 cases with discrepant diagnoses between EC1 and EC2 were presented to EC3 to achieve consensus. In 77 of these cases, EC3 considered the diagnoses by the previous two physicians as sufficiently congruent (e.g., T14 = Injury of unspecified body region and T13 = Other injuries of lower limb, level unspecified). These ICD-10 codes are presented in S4 Table. Considering these congruent diagnoses, EC1 and EC2 agreed in 75.8% of the cases. In 43 cases, EC2 had coded rather symptoms than a diagnosis (e.g., R10 = abdominal and pelvic pain and R11 = nausea and vomiting as symptoms of A09 = infectious gastroenteritis and colitis, unspecified) while EC1 used to code the overall complaint (comprising the symptoms coded by EC2). These cases were marked as “matching symptoms” by EC3 but not considered concurrent because the symptoms could occur with different diagnoses.

### Diagnostic accuracy

In 58.4% of cases, EC and OOHP records showed at least one identical diagnosis. This was the case in 57.6% of cases in the intervention group and 59.1% of cases of the control group. We assessed the intervention effect with two logistic mixed-models (as described in the statistics section) (Table 3). None of the models showed a significant intervention effect. The only significant variable was the number of diagnoses (p = 0.01 in model 2). Subgroup analyses can be found in S5 Table and the agreement between physicians separately for both centers in S6 Table.

**Table 3:** Mixed-effect logistic regression on intervention effect for diagnostic accuracy adjusted for various variables.

	Model 1		Model 2	
	OR (95% CI)	p	OR (95% CI)	p
Intervention^1^	0.94 (0.73 – 1.21)	0.609	0.771 (0.55 – 1.08)	0.130
Center Northeim^2^	1.07 (0.83 – 1.39)	0.582	0.984 (0.67 – 1.49)	0.935
Patient age	–	–	1.267 (1.07 – 1.50)	**0.005**
Number of diagnoses	–	–	1.291 (1.01 – 1.65)	0.043
General practitioner^3^	–	–	0.764 (0.52 – 1.13)	0.181
Number of complaints	–	–	0.899 (0.79 – 1.03)	0.125
severity: mild^4^	–	–	1.054 (0.55 – 2.04)	0.875
severity: medium^4^	–	–	1.044 (0.57 – 1.90)	0.889
severity: severe^4^	–	–	2.955 (1.49 – 5.86)	**0.002**
severity: unbearable^4^	–	–	2.651 (0.55 – 12.80)	0.225

bold: p < 0.05; OR>1 indicates a higher chance of diagnostic accuracy in at least one diagnosis between OOHP and EC.

^1^Ref. Control group; ^2^ Ref. Center Göttingen; ^3^ Ref. not general practitioner; ^4^ Ref. no symptoms.

### Comparison of the recommendation for further treatment with the actual treatment

**Fig 5:**
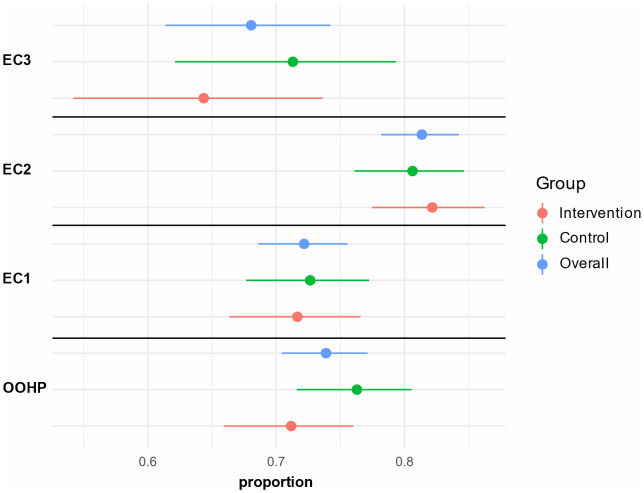
Agreement of further treatment of the patients stratified after physicians and group. Proportion and 95%-Confidence Interval. EC: expert committee physician; OOHP: out-of-hours practice physician.

The agreement with the actual further treatment of the patients stratified according to the assessing physicians and the group allocation is shown in Fig 5. We observed the lowest level of agreement with EC3 and the highest with EC2.

Identical to the primary outcome, no significant effect of the intervention on predicting further treatment was found in any of the models. The number of diagnoses reported by the OOHP and a self-reported severe condition were the only significant variables of interest in the models (Table 4).

**Table 4:** Mixed-effect logistic regression on intervention effect for accuracy on further treatment adjusted for various variables.

	Model 1		Model 2		Model 3		Model 4	
	OR (95% CI)	p	OR (95% CI)	p	OR (95% CI)	p	OR (95% CI)	p
Intervention	0.89 (0.65 – 1.22)	0.466	0.88 (0.64 – 1.21)	0.421	0.87 (0.64 – 1.20)	0.407	0.83 (0.60 – 1.15)	0.268
Center (Northeim)	1.258 (0.92 – 1.72)	0.153	1.072 (0.77 – 1.49)	0.677	1.01 (0.70 – 1.46)	0.956	1.03 (0.71 – 1.50)	0.883
Number of diagnoses	–	–	1.324 (1.05 – 1.66)	**0.016**	1.321 (1.05 – 1.66)	**0.017**	1.28 (1.01 – 1.62)	**0.046**
General Practitioner	–	–	–	–	0.871 (0.60 –1.27)	0.474	0.84 (0.57 – 1.23)	0.375
Number of complaints	–	–	–	–	–	–	0.92 (0.81 – 1.04)	0.170
severity: mild	–	–	–	–	–	–	1.10 (0.58 – 2.07)	0.771
severity: medium	–	–	–	–	–	–	1.13 (0.64 – 2.01)	0.668
severity: severe	–	–	–	–	–	–	3.16 (1.64 – 6.08)	**0.001**
severity: unbearable	–	–	–	–	–	–	2.723 (0.57 – 13.01)	0.209

bold: p < 0.05; in model 2, 3 and 4 all effects are further adjusted for patient age.

The agreement between physicians regarding the recommendation for further treatment overall and separately for both centers is shown in S7 Table.

## Discussion

This study examined whether a newly developed medical history-taking app affects diagnostic accuracy and treatment prediction in German out-of-hour practices. The app generates structured reports from patient inputs for physicians to review before consultation. Our assumption was that the app could be a valuable tool for the specific setting of OOHP – with limited time and diagnostic capacities, frequently changing staff, different medical specialties and nearly exclusively first-contact physician-patient encounters.

The medical history app did not significantly improve diagnostic accuracy (57.6% in the intervention vs 59.1% in the control group) or prediction of further treatment needs in OOHP. The only significant factors affecting those outcomes were the number of diagnoses (positively associated with diagnostic accuracy) and a self-reported severe condition (associated with higher likelihood of requiring further treatment).

While diagnostic accuracy is an established outcome in general practice research, it has mainly been studied in the context of diagnostic support systems that can suggest possible diagnoses and assist with symptom coding during consultations. In experimental studies, these decision support systems have been shown to increase diagnostic accuracy by 6 [27] to 9 [28] percentage points. A study simulating virtual groups found that pooling independent diagnoses from multiple GPs improved diagnostic accuracy [29]. Our study differs from these support system studies, as our app neither provides diagnostic suggestions nor relies on physician-entered data, but rather on patient-reported symptoms. However, future research could compare diagnostic suggestions based on patient-entered versus physician-entered data.

A finding in this study was the rather high agreement rate of EC and OOHP physicians. This finding is notable given that agreement rates between providers’ assessments have been shown to be low across various medical fields. For instance, a retrospective study found only 23.2% and 15.7% diagnostic agreement between primary care and hospital diagnoses for schizophrenia and bipolar disorder, respectively [30]. In breast pathology, three experienced breast pathologists achieved 59.8% of agreement after independent assessment of the cases [31]. A study on the classification of shoulder problems found only moderate agreement between the assessments of two physiotherapists’ who had taken medical history and performed a physical examination on the same patients [32]. Also, in many cases where patients seek a second opinion on their diagnosis or proposed plan of care, there is no consensus among physicians evaluating the same medical problem [33]. Agreement between EC1 and EC2 was higher (75.8%) than with OOHP physicians, yet even with identical clinical information, physicians’ interpretations varied. These differences likely stem from individual training, expertise, and personal preferences, as described in other studies [34,35].

Our study’s observations suggest that individual differences between physicians influence the measured diagnostic accuracy value: Specifically, the EC members employed distinct coding strategies when using ICD-10 to diagnose patients. One either coded symptoms associated with a potential diagnosis, the other coded the diagnosis itself, encompassing those symptoms. If we had considered these as congruent, agreement between EC1 and EC2 would have been nearly 5 percentage points higher. A systematic review of publications on the accuracy of diagnostic coding in the General Practice Research Database in the United Kingdom found that acute diagnoses were not as well recorded as chronic conditions [36]. Patients that took part in our study had acute complaints and diagnoses were almost exclusively on these acute conditions as these were the focus of the consultation. In some cases, chronic conditions were additionally coded by the OOHP physicians. However, there are no scientific studies on the coding behavior of physicians in primary care in Germany.

There are also variations in the accuracy of prediction of further treatment. When stratifying the accuracy of prediction of further treatment by physicians and groups, it became clear that the discrepancies between physicians were more pronounced than those between the intervention and control group.

The phenomenon of disagreement is thus not unique to this study. Our study suggests that a lack of consensus may be a common occurrence in medical practice in OOHP. It is essential to note that these disagreements do not necessarily indicate medical errors. Rather, they may reflect legitimate differences in coding behavior or professional opinion. In fact, these differences can sometimes lead to the same treatment decision.

### Strengths & limitations

Noteworthy, our study took place during a period of increased OOHP patient volume in 2022 [2], often resulting in full waiting rooms and the staff’s pressure to treat patients as quickly as possible. The study required additional time from both staff and patients; particularly the intervention group would jeopardize the practice schedule. This busy setting likely affected the intervention’s effectiveness. Under time pressure, physicians may have reverted to their individual history-taking routines rather than utilizing the unfamiliar app reports, potentially diminishing the intervention’s impact on diagnostic accuracy. However, we did not measure the extent to which physicians reviewed these reports (e.g., time spent on reviewing the summary) or how long the consultations took in the intervention vs. the control group. It was not possible to systematically monitor physicians’ engagement due to processes in the OOHP. In addition, limited technical skills may have hindered physicians to make adequate use of the app information. The OOHP used uncommon and outdated practice management software [37], which displayed only four lines of the app report at a time. This technical constraint limited physicians’ ability to review and utilize the app’s information effectively, potentially affecting diagnostic accuracy.

Other limitations include the operationalization of the outcome. Diagnostic accuracy operationalized using the ICD-code, even though a recognized outcome in medicine, might not be suitable for the OOHP setting. OOHP consultations primarily focus on assessing acute conditions [38] and determining immediate treatment paths – whether through hospital referral, prescription of a drug therapy or symptomatic relief. In the latter cases, these services mainly serve as a bridge to definitive treatment by the patient’s GP or other specialist care the following day or on Monday [39]. Therefore, the precise diagnosis is less critical than treatment decisions. The treatment decision can be the same for several different ICD-codes, though. This is in contrast to other areas of medicine, where objectively measurable parameters allow more definitive diagnoses to be made that may define a reference standard [40]. Alternatively, simulated patients and case vignettes with known diagnoses could have provided a more controlled evaluation. However, this approach would have sacrificed real-world validity and likely introduced its own biases in assessing the app’s practical effectiveness.

Related to the study design, a timing-related bias may have been introduced: intervention group patients, who used the app pre-consultation, consistently reported more complaints and higher burden than control group patients who used it post-consultation. This may indicate that the OOHP physician was influencing the control sample, e.g., by providing reassurance that complaints were not concerning. Therefore, control group patients may have omitted or recategorized complaints when using the app after the consultation. Consequently, the EC’s judgment is based on information of different situations, potentially influencing their diagnostic conclusions. In real-life, medical history-taking apps are used prior to consultation; the control group’s post-consultation app usage represents an artificial study condition that is unlikely to occur in practice. During the planning phase, we assumed that the process of structured self-assessment of complaints would help patients to articulate their complaints more effectively, improving the consultation and diagnostic accuracy.

The diagnoses coded in the OOHP are not necessarily limited to the diagnosis for the complaint that brought the patient to the OOHP. A previously known diagnosis may have been documented and coded (e.g., essential primary hypertension). Therefore, the ICD-10 coding may not be the correct measure. If the method of measurement is not appropriate, this may lead to inaccurate results. We cannot compare the chosen outcome measure with other validated methods, or with the methods used in previous studies to assess the validity of our chosen approach because there are no studies of this type.

The 2022 pandemic conditions may limit our study’s generalizability. OOHPs handle diverse conditions with abdominal and pelvic pain, back pain, minor injuries, and acute infectious conditions as the most common [38]. Pandemic protocols including minimal contact consultations (e.g., through a window) and expedited treatment for respiratory cases, are likely to have reduced the proportion of respiratory infections in our study population.

Our study is the first to evaluate the impact of a medical history app on diagnosis accuracy and treatment prediction in the real world. Given the lack of established or standardized approaches for evaluating medical history-taking apps, our attempt at implementation and evaluation in real-world settings can be considered a strength. Our experience highlights the importance of considering the complexities of study conduction in a real-life setting. Another strength is our long, one year-round data collection period with real world data in two centers.

The study’s limitations highlight the need for further research in this area. For future studies in OOHP, we suggest exploring other potential benefits of such approaches, such as the usefulness of the app from the physicians’ perspective (e.g., support for record-keeping, time savings, facilitated communication between healthcare providers), patient satisfaction and engagement, its impact on health outcomes, and in different healthcare setting (e.g., emergency scenarios, including pre-hospital emergency care).

## Conclusions

The study’s results suggest that the app did not improve diagnostic accuracy in a real-life setting. Future studies can explore other potential benefits of medical history apps on health outcome and explore other potentials for the quality of care. These may include the app’s primary purpose in facilitating medical history-taking and documentation.

## Supporting information

S1 Table Characteristic of participants included and excluded from expert committee and statistical analyses. (DOCX)

S2 Table Data cleansing. (DOCX)

S3 Table Complaints stated by participants. Not chosen: Fainting or blacking out.
(DOCX)

S4 Table Diagnoses considered being sufficiently congruent. (DOCX)

S5 Table Subgroup analyses: Mixed-effect logistic regression on intervention effect for diagnostic accuracy. Bold: p < 0.05; OR>1 indicates a higher chance of diagnostic accuracy in at least one diagnosis between OOHP and EC. GP: general practitioner.
(DOCX)

S6 Table Agreement of expert committee’s diagnoses and the OOHP physicians diagnoses per center. (DOCX)

S7 Table Agreement between recommendations of further treatment between physicians. Data is proportion in % (95% CI). EC: expert committee physician; OOHP: out-of-hours practice physician.
(DOCX)

S1 Data Minimal data set. (XLSX)
