# Supplementary material for: Taking a closer look: Can an app improve diagnostic accuracy in urgent care? Cluster-randomized interventional trial DASI
Source: PLOS Digit Health. 2026 Feb 24;5(2):e0001252. doi: 10.1371/journal.pdig.0001252 (PMC12931775; doi:10.1371/journal.pdig.0001252)
Supplement: S1 Table — (DOCX) [file pdig.0001252.s001.docx]

**S1 Table. Characteristic of participants included and excluded from expert committee and statistical analyses.**

|  |  | **In expert committee** | **Excluded from expert committee** | **p** |
| --- | --- | --- | --- | --- |
| N |  | 986 | 54 |  |
| Sex (n (%)) | male | 384 (38.9) | 27 (50.0) | 0.140 |
|  | female | 602 (61.1) | 27 (50.0) |  |
| age (median [IQR]) | years | 31.00 [24.00, 44.00] | 38.00 [30.50, 52.75] | 0.001 |
| Number of diagnoses (median [IQR]) | n | 1.00 [1.00, 2.00] | 1.00 [1.00, 1.00] | 0.211 |
| height (median [IQR]) | cm | 171.00 [165.00, 180.00] | 175.00 [168.00, 181.50] | 0.055 |
| weight (median [IQR]) | kg | 77.00 [65.00, 90.00] | 83.00 [72.75, 95.00] | 0.095 |
| Highest professional qualification (n [%]) | Master’s degree/Diploma/State examination/ PhD | 128 (13.1) | 12 (22.2) | 0.101 |
|  | Bachelor‘s degree | 78 (8.0) | 4 (7.4) |  |
|  | Master craftsman/technician or equivalent | 39 (4.0) | 3 (5.6) |  |
|  | Completed vocational training | 240 (24.6) | 8 (14.8) |  |
|  | High school diploma / Advanced technical college certificate | 214 (21.9) | 10 (18.5) |  |
|  | Secondary school certificate | 168 (17.2) | 9 (16.7) |  |
|  | Elementary/lower secondary school certificate | 84 (8.6) | 4 (7.4) |  |
|  | Other qualifications | 11 (1.1) | 3 ( 5.6) |  |
|  | No degree | 13 (1.3) | 1 (1.9) |  |
| Main employment status (n [%]) | Employed/working | 531 (55.5) | 32 (61.5) | 0.571 |
|  | In vocational training | 84 (8.8) | 1 (1.9) |  |
|  | University student | 154 (16.1) | 6 (11.5) |  |
|  | Voluntary/civilian/military service | 6 (0.6) | 0 (0.0) |  |
|  | Student at school | 22 (2.3) | 2 (3.8) |  |
|  | Retired | 59 (6.2) | 4 (7.7) |  |
|  | Unemployed | 18 (1.9) | 2 (3.8) |  |
|  | other | 83 (8.7) | 5 (9.6) |  |
| German as native language (n (%)) | Yes | 856 (87.9) | 50 (92.6) | 0.409 |
| Recruitment center  (n [%]) | Northeim | 445 (45.1) | 20 (37.0) | 0.306 |
| Group  (n [%]) | Intervention group | 472 (47.9) | 30 (55.6) | 0.337 |
