# Supplementary material for: Taking a closer look: Can an app improve diagnostic accuracy in urgent care? Cluster-randomized interventional trial DASI
Source: PLOS Digit Health. 2026 Feb 24;5(2):e0001252. doi: 10.1371/journal.pdig.0001252 (PMC12931775; doi:10.1371/journal.pdig.0001252)
Supplement: S2 Table — (DOCX) [file pdig.0001252.s002.docx]

**S2 Table. Data cleansing.**

| **Missing data records, attributed to:** |
| --- |
| - Missing paper-pencil questionnaires from physicians who did not support the study - Paper-pencil questionnaires unintentionally not completed or not completed promptly by physicians - Missing printouts of patient documentation from study participants treated by physicians who did not support the study - Missing printouts of patient documentation due to other reasons (e.g. printer issues) - Lack of app data of control group participants who left the practice early for various reasons |
| **Discrepancies in the course of the plausibility check, which led to exclusion from the evaluation by the expert committee:** |
| - Participants who, according to patient documentation, did not visit the practices because of acute complaints (but, for example, to change dressings, to obtain a prescription or to extend a certificate of incapacity for work) - Different diagnoses in the physician's questionnaire and on the printed patient documentation which suggests a swapping of IDs / patients - Errors in the implementation of the intervention (app data not transferred correctly or not at all into the electronic medical record before the medical consultation and were therefore not available to the physicians) - Obvious swapping of IDs or group assignment |
