# Supplementary material for: Taking a closer look: Can an app improve diagnostic accuracy in urgent care? Cluster-randomized interventional trial DASI
Source: PLOS Digit Health. 2026 Feb 24;5(2):e0001252. doi: 10.1371/journal.pdig.0001252 (PMC12931775; doi:10.1371/journal.pdig.0001252)
Supplement: S3 Table — Not chosen: Fainting or blacking out. (DOCX) [file pdig.0001252.s003.docx]

**S3 Table. Complaints stated by participants.**

| **Complaint** | **n** |
| --- | --- |
| Sore throat | 147 |
| Abdominal discomfort | 105 |
| Cough | 105 |
| Headache | 100 |
| Difficulty swallowing | 99 |
| Back pain | 85 |
| Urinary discomfort | 84 |
| Cold or flu | 80 |
| Swelling | 80 |
| Changes in skin, hair or nails | 79 |
| Chest and heart problems | 73 |
| Joint and muscle problems | 71 |
| Ear problems | 71 |
| Insect bites or string | 65 |
| Injury | 64 |
| Dizziness | 50 |
| Shortness of breath | 43 |
| Weaknesses | 41 |
| Diarrhoea | 41 |
| Vomiting / nausea | 41 |
| Leg pain/leg swelling | 40 |
| Sleep disturbances or fatigue | 31 |
| Eye problems | 28 |
| Palpitations | 27 |
| Anxiety | 19 |
| Groin problems | 18 |
| Sweating | 16 |
| Fever | 14 |
| Changes in bowel movements / constipation | 13 |
| Depression | 10 |
| Forgetfulness | 9 |
| Anus problems | 7 |
| Weight changes | 7 |
| Paralysis/ sensory problems | 7 |
| Bleeding | 6 |
| Unusual vaginal bleeding | 6 |
| Unusual vaginal discharge | 5 |

Not chosen: Fainting or blacking out.
