# Supplementary material for: Taking a closer look: Can an app improve diagnostic accuracy in urgent care? Cluster-randomized interventional trial DASI
Source: PLOS Digit Health. 2026 Feb 24;5(2):e0001252. doi: 10.1371/journal.pdig.0001252 (PMC12931775; doi:10.1371/journal.pdig.0001252)
Supplement: S4 Table — (DOCX) [file pdig.0001252.s004.docx]

**S4 Table. Diagnoses considered being sufficiently congruent.**

| G44, R51 |
| --- |
| H10, H11 |
| H60, H61 |
| J02, J04, J06, J15, J18, J20 |
| K05, K12 |
| K58, K59 |
| K61, L02 |
| K62, K64 |
| K80, K82 |
| L03, S61 |
| L05, L08, L30, R21 |
| L23, L25, R21 |
| M19, M25 |
| M53, M54, M79, M99 |
| N02, R31 |
| N23, N30, N32, N39 |
| N50, N51 |
| N61, N64, O91 |
| R22, S60 |
| S50, S60, S70, S80, S90, S93, T00, T11, T13, T14 |
| S70, S81, T13, T89 |
| T22, X19 |
| T79, T89 |
