# Supplementary material for: Taking a closer look: Can an app improve diagnostic accuracy in urgent care? Cluster-randomized interventional trial DASI
Source: PLOS Digit Health. 2026 Feb 24;5(2):e0001252. doi: 10.1371/journal.pdig.0001252 (PMC12931775; doi:10.1371/journal.pdig.0001252)
Supplement: S5 Table — Bold: p < 0.05; OR>1 indicates a higher chance of diagnostic accuracy in at least one diagnosis between OOHP and EC. GP: general practitioner. (DOCX) [file pdig.0001252.s005.docx]

**S5 Table. Subgroup analyses: Mixed-effect logistic regressions on intervention effect for diagnostic accuracy.**

|  | **German as native language** | | | | **Year of state examinations** | | | | | | **Patient age** | | | | **Number of complaints** | | | |
| --- | --- | --- | --- | --- | --- | --- | --- | --- | --- | --- | --- | --- | --- | --- | --- | --- | --- | --- |
|  | Yes | | No | | Before 1990 | | 1990-2000 | | After 2000 | | <60 years | | ≥60 | | 1 | | 2-12 | |
|  | OR (95% CI) | p | OR (95% CI) | p | OR (95% CI) | p | OR (95% CI) | p | OR (95% CI) | p | OR (95% CI) | p | OR (95% CI) | p | OR (95% CI) | p | OR (95% CI) | p |
| Intervention^1^ | 0.87 (0.66–1.14) | 0.322 | 1.31 (0.62–2.8) | 0.484 | 0.90 (0.48–1.70) | 0.742 | 1.044 (0.75–1.46) | 0.803 | 1.02 (0.55–1.90) | 0.957 | 0.99 (0.76–1.29) | 0.919 | 0.55 (0.23–1.23) | 0.170 | 0.80 (0.59–1.10) | 0.177 | 1.27 (0.82–1.96) | 0.277 |
| Center Northeim^2^ | 1.17 (0.89–1.54) | 0.258 | 0.72 (0.33–1.55) | 0.399 | 0.90 (0.47–1.70) | 0.739 | 1.041 (0.73–1.48) | 0.823 | 1.44 (0.78–2.67) | 0.241 | 1.13 (0.86–1.48) | 0.373 | 0.58 (0.23–1.44) | 0.237 | 0.77 (0.56–1.05) | 0.099 | 1.98 (1.27–3.10) | **0.003** |

Bold: p<0.05; OR>1 indicates a higher chance of diagnostic accuracy in at least one diagnosis between OOHP and EC.

|  | **Severity** | | | | | | | | | | **Physician is GP** | | | | **Center** | | | |
| --- | --- | --- | --- | --- | --- | --- | --- | --- | --- | --- | --- | --- | --- | --- | --- | --- | --- | --- |
|  | [0] I don’t feel sick | | [1] Mild | | [2] Medium | | [3] Severe | | [4] Unbearable | | yes | | no | | 1 | | 2 | |
|  | OR (95% CI) | p | OR (95% CI) | p | OR (95% CI) | p | OR (95% CI) | p | OR (95% CI) | p | OR (95% CI) | p | OR (95% CI) | p | OR (95% CI) | p | OR (95% CI) | p |
| Intervention^1^ | 0.40 (0.18–0.88) | **0.023** | 0.79 (0.43–1.43) | 0.438 | 1.16 (0.81– 1.67 | 0.422 | 1.17 (0.65–2.11) | 0.597 | 0.00 (0.00–0.16) | **0.027** | 1.04 (0.76–1.43 | 0.792 | 0.77 (0.50– 1.19) | 0.240 | 1.00 (0.71–1.41) | 0.982 | 0.86 (0.59–1.25) | 0.431 |
| Center Northeim^2^ | 0.88 (0.40–1.97) | 0.763 | 1.55 (0.86–2.81) | 0.146 | 0.974 (0.68–1.40) | 0.888 | 1.01 (0.56–1.8) | 0.981 | 0.40 (0.00– 6,756) | 0.780 | 1.07 (0.75–1.51) | 0.721 | 1.19 (0.73– 1.96) | 0.484 | - | - | - | - |

Bold: p<0.05; OR>1 indicates a higher chance of diagnostic accuracy in at least one diagnosis between OOHP and EC. GP: general practitioner.
