# Supplementary material for: Taking a closer look: Can an app improve diagnostic accuracy in urgent care? Cluster-randomized interventional trial DASI
Source: PLOS Digit Health. 2026 Feb 24;5(2):e0001252. doi: 10.1371/journal.pdig.0001252 (PMC12931775; doi:10.1371/journal.pdig.0001252)
Supplement: S6 Table — (DOCX) [file pdig.0001252.s006.docx]

**S6 Table. Agreement of expert committee’s diagnoses and the OOHP physicians diagnoses per center.**

|  |  | **Center 1** | **Center 2** |
| --- | --- | --- | --- |
| EC1 | EC2 | 78.9% | 79.8% |
| EC1 | EC3 | 78.4% | 80.0% |
| EC1 | OOHP | 55.6% | 58.7% |
| EC2 | EC3 | 71.6% | 73.6% |
| EC2 | OOHP | 59.7% | 57.8% |
| EC3 | OOHP | 52.3% | 52.1% |

EC: expert committee physician; OOHP: out-of-hours practice.
