# Supplementary material for: Taking a closer look: Can an app improve diagnostic accuracy in urgent care? Cluster-randomized interventional trial DASI
Source: PLOS Digit Health. 2026 Feb 24;5(2):e0001252. doi: 10.1371/journal.pdig.0001252 (PMC12931775; doi:10.1371/journal.pdig.0001252)
Supplement: S7 Table — Data is proportion in % (95% CI). EC: expert committee physician; OOHP: out-of-hours practice physician. (DOCX) [file pdig.0001252.s007.docx]

**S7 Table. Agreement between recommendations of further treatment between physicians (both centers).**

|  | EC1 | EC2 | EC3 |
| --- | --- | --- | --- |
| OOHP | 70.2 (67.2 – 73.0) | 72.8 (70.0 – 75.6) | 66.1 (60.1 – 71.3) |
| EC1 | - | 77.8 (75.1 – 80.4) | 76.3 (71.2 – 80.8) |
| EC2 | - | - | 76.3 (71.2 – 80.8) |

Data is proportion in % (95% CI). EC: expert committee physician; OOHP: out-of-hours practice physician.

The agreement between the physicians on the recommendation for further treatment varied between 66.1% and 77.8%. The lowest concordance was between the third EC physician and the physician in the OOHP. The highest agreement was between the EC physician 1 and 2.

**Agreement between recommendations of further treatment between physicians (center 1).**

|  | EC1 | EC2 | EC3 |
| --- | --- | --- | --- |
| OOHP | 71.1 (0.67 – 0.75) | 75.3 (0.72 – 0.79) | 68.2 (0.61 – 0.75) |
| EC1 | - | 75.6 (0.72 – 0.79) | 75.0 (0.68 – 0.81) |
| EC2 | - | - | 73.3 (0.66 – 0.80) |

Data is proportion in % (95% CI). EC: expert committee physician; OOHP: out-of-hours practice physician.

The agreement between the physicians on the recommendation for further treatment varied between 68.2% and 75.6% for center 1. The lowest concordance was between the third EC physician and the physician in the OOHP. The highest agreement was between the EC physician 1 and 2.

**Agreement between recommendations of further treatment between physicians (center 2).**

|  | EC1 | EC2 | EC3 |
| --- | --- | --- | --- |
| OOHP | 69.2 (0.65 – 0.73) | 69.8 (0.65 – 0.74) | 63.6 (0.55 – 0.71) |
| EC1 | - | 80.4 (0.77 – 0.84) | 77.9 (0.70 – 0.84) |
| EC2 | - | - | 80.0 (0.72 – 0.86) |

Data is proportion in % (95% CI). EC: expert committee physician; OOHP: out-of-hours practice physician.

The agreement between the physicians on the recommendation for further treatment varied between 63.6% and 80.4% for center 2. The lowest concordance was between the third EC physician and the physician in the OOHP. The highest agreement was between the EC physician 2 and the physician in the OOHP.
